# Areas of work-life in Spanish hostelry professionals: explanatory power on burnout dimensions

**DOI:** 10.1186/s12955-019-1201-2

**Published:** 2019-07-30

**Authors:** Santiago Gascón, Bárbara Masluk, Jesús Montero-Marin, Michael P. Leiter, Paola Herrera, Agustín Albesa

**Affiliations:** 1 Department of Psychology and Sociology, University of Zaragoza, Zaragoza, Spain; 2 Primary Care Prevention and Health Promotion Research Network (RedIAPP), Zaragoza, Spain; 3 School of Psychology, Deakin University, Geelong, VIC Australia

**Keywords:** Areas of work-life, AWL, Engagement, Tourism professionals, Explanatory power

## Abstract

**Background:**

Researchers have studied for decades workplace stress and burnout to identify their relationship to health and wellness. This research has focused on stress levels in people, as well as on environmental and personal factors that contribute to experiencing stress or burnout. In addition to the burnout measurement questionnaires (MBI-GS), Leiter and Maslach designed a model to evaluate the areas of work environment that relate to this construct (Areas of Worklife Scale-AWLS).

The goal of the present research was to analyze the psychometric properties of a Spanish translation of the MBI (GS) and the AWLS with a Spanish-speaking population. This work makes a substantial contribution by addressing the need to use validated measures and methods when exploring the positive and negative aspects of organizations. These conditions provide a means to accurately evaluate the impact of interventions aimed to address stress and burnout.

**Method:**

Cross-sectional study with self-report measures. The sample was comprised of 452 managers and employees (hotels, restaurants, catering) of Aragón (Spain). There were approximately equal numbers of women and men (45,4% vs. 54,6%). The average age of participants was 36.6 years (SD = 10.03). A battery of questionnaires was used: *Socio-demographic and work characteristics*, *Scale of stress and health symptoms, Maslach Burnout Inventory-General Survey* (MBI-GS), *Areas of Worklife Scale (*AWLS).

**Results:**

The results showed optimal psychometric properties in both questionnaires, especially in terms of the predictive capacity of the AWLS in each of the MBI-GS dimensions.

**Conclusions:**

The best explained dimension is that of emotional exhaustion. The manageable load variable is the one that most contributes to predicting burnout levels. For future interventions, the results confirm the need to verify the levels of each area of work, in order to focus on the most deteriorated ones.

## Introduction

The concept of burnout, coined by Freudenberger [1], refers to a crisis in the employees’ work experience. Over the following decades there has been controversy about whether burnout was a depressive state of occupational origin [2], a unidimensional construct characterized by the feeling of exhaustion [3], or a complex syndrome [4]. As a chronic condition, has been shown to have consequences for the workers’ health: anxiety, depression and cardiovascular, dermatological, digestive, immunological problems, among others). It can also have negative consequences for the enterprise - higher absenteeism rate, sick leave or job abandonment [5].

Maslach & Jackson [4] created the “Maslach Burnout Inventory” (MBI) as a measurement instrument, with three subscales corresponding to emotional exhaustion, depersonalization and lack of personal accomplishment. For more than four decades, such conceptualized burnout syndrome has led to countless studies across occupations and countries [6, 7]. However, since its original publication, some of its limitations and shortcomings have also been evident, which has promoted the development of new inventories for the measurement of burnout from other theoretical perspectives [8, 9].

Maslach and Leiter [10] proposed a turnabout in research based on the “engagement” construct, a concept that could be considered as the opposite of burnout, with positive experiences of energy, involvement, and effectiveness. They argued that burnout and engagement described opposite ends of a continuum that could be measured with a questionnaire. This was the purpose of the most recent version of the “Maslach Burnout Inventory - General Survey” (MBI-GS) [11, 12], which considers the dimensions of exhaustion, cynicism and professional efficacy. Burnout is indicated by negative scores on all three dimensions and engagement is indicated by positive scores. Other developments have defined work engagement with separate measures, most notably the Utrecht Work Engagement Scale [13]. Nevertheless, burnout and engagement seem to be concepts that overlap to some extent, as it can be seen in studies such as the meta-analysis from Rich, Lepine and Crawford [14].

In order to advance the knowledge of this construct, Leiter and Maslach [15] proposed a model based on variables of the Areas of Work Life Model (AWL), which contribute to burnout or engagement experiences. These areas are: workload, control, rewards, community, fairness and values, and are based on the findings of multiple studies on work-related stress.

Workload and control are consistent with the Demand-Control model of job stress [16]. Several studies have revealed that the increase in workload has a direct relationship with the dimension of exhaustion [17]. However, other variables can protect from exhaustion, even in high overload situations. A high index of task control by the workers could protect him from burnout, while a low level in this variable could increase the effects of overload [18]. The rewards variable or the reinforcement capacity when directing behavior is related to intrinsic rewards, to the satisfaction that comes from the recognition of a task well done [19]. While the community variable tries to reflect both supportive relationships and conflicts between workers [18], the area of justice is supported by the literature that develops the theory of equity in the work environment [20]. Employees with burnout specially caused by high perceptions of unfairness have been described by Leiter and Harvie [21]. Finally, the area of values refers to the congruence or conflict between the values of the individual and those of the organization, as well as the emotional and cognitive power of the workers’ goals and expectations with respect to the present situation [22].

The Areas of Work Life Model (AWLS) explains the processes by which a person can become worn out in their work environment. The employees of an organization can be exposed to high levels of overload, but the fact of feeling reinforced by some kind of reward, or by the good atmosphere among colleagues and/or managers, makes the negative workload effect on burnout being attenuated by these variables. Over time, these variables may not be as positive and they can even reach negative levels, aggravating the workload effects on burnout [18].

Studies from the AWLS model show that the six areas explain a significant percentage of the burnout variance in working populations from different countries and different economic sectors [23, 24].

This study was carried out in hostelry, since it is a representative working environment of the service sector in Spain [25].

Moreover, and due to the absence of studies on the psychometric characteristics of the AWLS measure in the specific context of hotel workers, we also aimed to evaluate the psychometric characteristics of the AWLS questionnaire among these professionals, as well as its relationships with general socio-demographic and work indicators, such as the level of absenteeism, the intention to stop working, and the general symptomatology associated with chronic stress situations among these hotel professionals.

## Method

This was a cross-sectional study with self-report measurements conducted in 5 hotels and 11 restaurants. Confirmatory Factor Analysis of AWLS was tested. Associations between socio-demographic and labour characteristics (as independent variables) and the AWLS factors (as dependent variables) were used with logistic regression (LR) models.

### Participants

The sample of participants was composed of 452 employees and managers of the service sector (hotels, restaurants and catering companies), purposively selected from a large chain of hotels and restaurants in the region of Aragón (Spain). We reached a sample size, which was estimated according to the evaluation criterion of the 10:1 recommended ratio for the number of subjects and number of test items included in the confirmatory factor analysis. Therefore, psychometric adequacy was provided to the analysis [26]. The inclusion criteria were: a) active work situation in hotels or restaurants, b) age between 18 and 65 years, c) being able to read Spanish.

### Procedure and ethics

The study responded to a call from the Government of Aragón, funded by the European Union, that had been evaluated beforehand and afterwards by these institutions in terms of its design, methodology, and ethical aspects. Contact was made with hotel and restaurant associations, who facilitated interviews with managers and human resources managers to obtain information on large chains, medium-sized hotels and different size restaurants. Informative talks were held with managers and employees in groups of 5 to 20 participants. These sessions provided information on the general characteristics of the study, emphasizing anonymous data protection and voluntary participation. The questionnaires were administered after obtaining the written informed consent of the participants.

### Measurements

#### Socio-demographic and work characteristics

Including sex, age, relationships (in a stable relationship, non-stable relationship), children (yes, no), type of company (hotel, restaurant), level of responsibility (employee, manager), contract type (permanent, temporary), number of sick leave days over the past year (this answer was contrasted with the data provided by each company), and intentions to stop working (yes, no).

#### Stress and health symptom scale

This self-made scale attempts to record the frequency at which the employee has experienced over the last 12 months symptoms such as ‘headaches’, ‘depressed mood’, ‘gastro-intestinal problems’, ‘perceived stress’ or ‘presence of infections’; as well as possible harmful behaviors such as the use of alcohol, tobacco, drugs, or being overweight. The answers show a Likert format, with a range between 0 (never) and 6 (always). The internal consistency of the state of stress symptoms in this sample was adequate, with a McDonald’s omega value of ω = 0.72.

#### Maslach burnout inventory-general survey (MBI-GS [12, 27]

It consists of 16 items that report on the three burnout dimensions ―e.g., exhaustion, cynicism, and (lack of) efficacy. Thus, for example, to the question “I feel emotionally exhausted at work”, the answer should inform of the frequency with which it happens, ranging from 0, never, to 6, daily. The questionnaire has shown a good factorial structure and adequate internal consistency [28]. The psychometric characteristics of the MBI-GS in the study sample are described in the results section.

#### Areas of Worklife scale (AWS) [18, 26]

It reports on the possible adjustment or misalignment of major variables or areas of work life that may contribute to work attrition, such as manageable load, controllability, rewards, sense of community, fairness, and congruence of values. The questionnaire requests an answer to the items with which the subject agrees, to some extent, in each of the statements. For example: in the statement “I don’t have time to do the work that must be done”, the subject could answer with 1 (Strongly disagree) to 5 (Strongly agree). The Spanish adaptation of the AWS has proven to be internally consistent and valid in terms of structure in Spanish health workers [29]. The psychometric characteristics of the AWS in the study sample will be described in the results section.

##### Data analysis

We examined the socio-demographic variables, as well as the items’ psychometric characteristics, by using descriptive statistics such as means, standard deviations, frequencies, percentages, skewness, kurtosis and discrimination coefficients.

We used confirmatory factor analysis (CFA) to evaluate the factor structures of the AWLS and MBI-GS. The KMO, determinant, Bartlett’s statistic and Mardia’s coefficient were estimated from polychoric matrices, which are specially advised for factor analysis when using ordinal variables [30]. The unweighted least squares (ULS) factor analysis was used for factor extraction, due to its robustness [31]. This procedure does not require any distributional assumptions, it usually converges because of its efficiency in terms of computation, and it tends to provide less biased estimates of the true parameter value. Finally, it works especially well with polychoric matrices [32].

From a general perspective, the goodness of fit of the models was examined by using the goodness of fit index (GFI), the adjusted goodness of fit index (AGFI), the root mean square of the standardized residuals (RMSR), the normed-fit-index (NFI), and Bollen’s relative-fit-index (RFI). GFI and AGFI refer to explained variance, and values ≥0.90 are considered acceptable [33]. SRMR is the standardized difference between the observed and the predicted covariance, and values ≤0.08 indicate a good fit [34]. NFI measures the proportional reduction in the adjustment function when going from null to the proposed model, and values ≥0.90 are considered acceptable [35]. RFI takes into account the discrepancy of the proposed model and the baseline model, and values that are close to 1 indicate a good fit [36]. From an analytical point of view, the standardized factor loadings, the uniqueness term for each item (δ), and the standardized inter-factor correlations were also examined.

The internal consistency of each factor was calculated by using McDonald’s Omega (ω), which can be interpreted as the square of the correlation between the scale score and the latent common variable to all the indicators [37]. The Omega index assumes that factor loadings can vary, and it also considers the item-specific measurement error. Thus, it provides a more realistic estimate of true reliability than classical Cronbach’s Alpha values, given the fact that both can be interpreted using the same threshold cut-off points.

We used the AWS factors as independent variables in multivariate linear regression models in order to assess their contribution to explaining exhaustion, cynicism and lack of professional efficacy as dependent variables. Previously, the association degree regarding all the constructs, by means of r coefficients, was evaluated. Standardized beta coefficients were used to assess the individual contribution of each factor, and the Wald test was used to evaluate their significance. Adjusted multiple determination coefficients (R^2^_y.123_) were calculated to observe their grouped explanatory power, and their significance was assessed by means of variance analysis. Partial correlation coefficients (R_y3.12_) were also calculated. We discarded possible problems of collinearity by ensuring that tolerance values (Tol) and the variance inflation factor (VIF) did not exceed critical values (Tol < 0.10, VIF > 10) [38].

Finally, we also explored the associations between socio-demographic and work characteristics (as independent variables) and the AWLS factors (as dependent variables) using logistic regression (LR) models. In the absence of previously established cut-off points, it was suggested that the high scoring subjects would be those above the third quartile (25% of subjects) for each of the AWLS dimensions [39]. In the bivariate analysis, the possible association between the high/low levels of AWLS factors with each of the variables of interest was evaluated by means of a simple LR, which provided a raw odds ratio (OR), and its 95% confidence interval (CI) estimation. Factors that provided a statistically significant result in the bivariate analysis (*p* < 0.05) were then included in a multivariate LR model.

All the tests were bilateral and were performed with a significance level of *p* < 0.05. Data analyses were performed with the SPSS-19 and Amos-7 statistical software packages.

## Results

### Psychometric features of the MBI-GS and AWS scale

The correlation matrix of the MBI items showed adequate characteristics (Mardia’s = 36.05; *p* < 0.001; KMO = 0.89; Bartlett χ^2^ = 3,9828.40; df = 120; *p* < 0.001; determinant < 0.001). Table 1 shows the psychometric features of the items and factors from the burnout scale. All the standardized loadings of the items were appropriated (the item n° 5 was the lowest with a 0.41 value). The three-correlated factor solution explained a large amount of the variance (67.04%) and presented a very good fit to the data (GFI = 0.968; AGFI = 0.957; NFI = 0.951; RFI = 0.942; SRMR = 0.075). All the internal consistence values of composite reliability were very good (energy: ω = 0.93; involvement ω = 0.88; efficacy ω = 0.80). The inter-factor latent correlations between the MBI-GS dimensions were: exhaustion <− > cynicism *r* = 0.85; exhaustion <− > inefficacy *r* = 0.12; cynicism <− > inefficacy *r* = 0.27.

**Table 1:** Descriptive and factor loadings for the MBI-GS Scale

Factors / items	ω	Mn	SD	skew	kurt	item-r	λ	δ
Exhaustion (0–30)	0.93	8.80	7.46					
Emotional damage		1.92	1.68	−0.76	− 0.33	0.85	0.87	0.13
Feelings after work		2.16	1.73	−0.68	− 0.45	0.75	0.73	0.27
Low energy level		1.72	1.72	−1.12	0.42	0.80	0.84	0.16
Work as an effort		1.06	1.65	−1.70	1.97	0.74	0.83	0.17
Burned out		1.94	1.89	−1.62	1.62	0.75	0.83	0.17
Cinicism (0–30)	0.88	7.58	7.13					
Low interest		1.10	1.69	1.62	1.64	0.74	0.84	0.16
Low enthusiasm		1.47	1.71	1.18	0.39	0.67	0.79	0.21
Low commitment		2.54	2.24	0.37	−1.35	0.39	0.44	0.56
No significance		1.25	1.78	1.36	0.72	0.72	0.79	0.21
More cynical		1.21	1.80	1.43	0.96	0.68	0.74	0.26
Efficacy (0–36)	0.80	25.00	6.79					
Effectiveness		4.19	1.81	−0.91	−0.10	0.43	0.41	0.59
Contribution		4.36	1.68	−1.02	0.33	0.55	0.56	0.44
Self-evaluation		4.52	1.58	−1.13	0.77	0.56	0.60	0.40
Exhilaration		3.69	1.89	−0.43	−0.90	0.49	0.72	0.28
Accomplishment		3.50	1.79	−0.38	− 0.78	0.43	0.57	0.43
Self-confidence		4.73	1.49	−1.26	1.14	0.44	0.51	0.49

Range of factors in brackets

*Mn* mean, *SD* standard deviation, *Skew* skewness, *Kurt* kurtosis, *Item-r* ítem-rest coefficient

ω = McDonald’s Omega, λ = standardized loadings. δ = uniqueness

The correlation matrix of the AWLS showed adequate properties (Mardia’s = 46.29; *p* < 0.001; KMO = 0.86; Bartlett χ^2^ = 4,442.20; df = 406; *p* < 0.001; determinant < 0.001). Table 2 shows the psychometric features of the items and factors of the AWS scale. In general, all the standardized loadings of the items were adequate (although item n° 22 showed a value of 0.26). The six-correlated factor solution explained an important amount of the variance (53.83%) and presented a good fit to the data (GFI = 0.942; AGFI = 0.930; NFI = 0.905; RFI = 0.893; SRMR = 0.071). All the internal consistence values of composite reliability were adequate, ranging from ω = 0.68 (control) to ω = 0.78 (reward and fairness). As it can be seen in Table 3, the inter-factor latent correlations between the AWLS dimensions ranged from *r* = 0.32 (overload <− > control) to *r* = 0.81 (justice <− > values).

**Table 2:** Descriptive and factor loadings for the AWLS

Factors / items	ω	Mn	SD	skew	kurt	item-r	λ	δ
Workload^a^ (5–30)	0.74	18.67	4.63					
No time to do work		3.39	1.22	−0.22	−0.93	0.49	0.52	0.48
Prolonged periods		2.51	1.19	0.56	−0.66	0.37	0.36	0.64
Too tired after work		2.81	1.30	0.17	−1.14	0.52	0.61	0.39
No personal interest		3.24	1.23	−0.14	−0.94	0.53	0.79	0.21
Enough time		3.43	1.10	−0.53	−0.36	0.36	0.55	0.45
Work left behind		3.29	1.40	−0.28	−1.22	0.23	0.26	0.74
Control (3–15)	0.68	9.45	2.69					
Have control		3.48	1.18	−0.61	−0.39	0.46	0.53	0.47
Influence		3.08	1.22	−0.29	−0.87	0.47	0.60	0.40
Autonomy		2.90	1.16	−0.08	−0.82	0.39	0.67	0.33
Reward (4–20)	0.78	13.09	3.44					
Recognition		3.13	1.19	−0.40	−0.80	0.59	0.74	0.26
Appreciated		3.53	0.99	−0.86	0.56	0.44	0.66	0.44
Unnoticed		3.19	1.18	−0.24	−0.90	0.64	0.67	0.33
Not recognized		3.25	1.18	−0.30	−0.80	0.51	0.57	0.43
Community (5–25)	0.77	17.69	3.92					
People trust		3.61	1.03	−0.77	0.33	0.49	0.62	0.38
Supportive group		3.47	1.15	−0.78	− 0.14	0.37	0.58	0.42
Cooperation		3.53	1.17	−0.74	− 0.17	0.63	0.73	0.27
Communication		3.45	1.17	−0.69	− 0.41	0.67	0.74	0.26
Not close to others		3.63	1.22	−0.63	− 0.55	0.26	0.30	0.70
Fairness (6–30)	0.78	18.44	4.77					
Resources		3.13	1.14	−0.42	−0.43	0.47	0.56	0.44
Opportunities		2.79	1.14	−0.06	−0.75	0.48	0.55	0.45
Appeal procedures		3.00	1.10	−0.26	−0.55	0.47	0.65	0.35
Fair treat		3.02	1.23	−0.25	−0.93	0.66	0.77	0.23
Favoritism		3.30	1.21	−0.24	−0.85	0.42	0.49	0.51
Acquaintance		3.19	1.31	−0.17	−1.10	0.46	0.49	0.51
Values (5–25)	0.77	17.55	3.77					
Similarity		3.08	1.15	−0.29	−0.64	0.48	0.67	0.33
Influence		3.60	1.02	−0.77	0.35	0.51	0.54	0.46
Consistency		3.09	1.14	−0.18	−0.64	0.58	0.70	0.30
Quality		3.78	1.10	−0.94	0.33	0.50	0.56	0.44
Commitment		4.00	1.07	−0.97	0.42	0.34	0.44	0.56

Range of factors in brackets

*Mn* mean, *SD* standard deviation, *Skew* skewness, *Kurt* kurtosis, *Item-r* ítem-rest coefficient

ω = McDonald’s Omega. λ = standardized loadings. δ = uniqueness

^a^Manageable workload

**Table 3:** Inter-factor latent correlations between the AWLS components

	1	2	3	4	5	6
1. Workload^a^	1					
2. Control	0.32	1				
3. Reward	0.50	0.56	1			
4. Community	0.33	0.55	0.64	1		
5. Fairness	0.38	0.62	0.64	0.65	1	
6. Values	0.45	0.65	0.67	0.58	0.81	1

Values are standardized correlations of latent variables

^a^Manageable workload

### Explanatory power of the AWLS on burnout

Tolerance values among the AWLS dimensions (all of them ≥0.55) and the VIF values (all of them ≤1.82) were adequate, thus no collinearity problems were observed. The explanatory power of the AWS dimensions on the burnout components was significant (Table 4). The most explained burnout dimension was exhaustion (adjusted *R*^*2*^ = 0.23; *p* < 0.001), whilst the least explained burnout dimension was cynicism (adjusted *R*^*2*^ = 0.08; *p* < 0.001), being efficacy in the middle (adjusted *R*^*2*^ = 0.18; *p* < 0.001). Exhaustion was explained by (lack of) manageable workload (Beta = − 0.41; *p* < 0.001). Cynicism was explained by (lack of) manageable workload (Beta = − 0.14; *p* = 0.007) and (lack of) reward (Beta = − 0.12; *p* = 0.041). Efficacy was explained by (lack of) manageable workload (Beta = − 0.19; *p* < 0.001), control (Beta = 0.14; *p* = 0.008), community (Beta = 0.25; *p* < 0.001), and values (Beta = 0.24; *p* < 0.001). The standard errors of the models ranged from 5.10 (efficacy) to 6.81 (cynicism).

**Table 4:** Explanatory power of the AWS on the engagement dimensions

Models/*variables*	R_y.123_	adj-R^2^_y.123_	F (df_1_ / df_2_)	*p*^a^	Se
Exhaustion	0.49	0.23	21.80 (6 / 418)	< 0.001	6.51
Cynicism	0.31	0.08	7.1 (6 / 418)	< 0.001	6.84
Efficacy	0.44	0.18	16.9 (6 / 418)	< 0.001	5.10
	r	R_y3.12_	B (95% CI)	Beta	*p*^b^
Exhaustion					
Workload^c^	−0.46	−0.40	−0.65 (−0.80 – −0.51)	−0.41	< 0.001
Control	−0.21	− 0.06	−0.17 (− 0.43–0.10)	−0.06	0.217
Reward	−0.24	− 0.01	−0.02 (− 0.25–0.21)	−0.01	0.887
Community	−0.21	− 0.04	−0.08 (− 0.27–0.11)	−0.04	0.410
Fairness	−0.25	− 0.07	−0.13 (− 0.30–0.05)	−0.08	0.159
Values	−0.24	− 0.02	−0.05 (− 0.28–0.17)	−0.03	0.635
Cynicism					
Workload^c^	−0.22	− 0.13	−0.21 (− 0.36 – − 0.06)	−0.14	0.007
Control	−0.15	− 0.03	−0.08 (− 0.36–0.20)	− 0.03	0.571
Reward	−0.25	− 0.10	−0.25 (− 0.49 – − 0.01)	−0.12	0.041
Community	−0.22	− 0.08	−0.17 (− 0.37–0.04)	−0.09	0.106
Fairness	−0.19	− 0.03	−0.07 (− 0.25–0.12)	−0.04	0.482
Values	−0.18	− 0.01	−0.01 (− 0.24–0.23)	−0.01	0.947
Efficacy					
Workload^c^	−0.07	−0.19	− 0.23 (− 0.34 – − 0.12)	− 0.19	< 0.001
Control	0.25	0.13	0.28 (0.08 – 0.49)	0.14	0.008
Reward	0.13	−0.09	− 0.16 (− 0.34 – 0.02)	− 0.05	0.076
Community	0.29	0.22	0.35 (0.20 – 0.50)	0.25	< 0.001
Fairness	0.22	0.01	0.01 (− 0.13 – 0.14)	0.01	0.953
Values	0.29	0.19	0.35 (0.17 – 0.52)	0.24	< 0.001

*R*_*y.123*_ multiple correlation coefficient, *Adj-R*^*2*^_*y.123*_ adjusted coefficient of multiple determination, *p*^*a*^ *p* value for variance analysis associated with the regression, *Se* standard error, *r* raw correlation coefficient, *R*_*y3.12*_ partial correlation coefficient, *B* regression slope, *95% CI* 95% confidence interval, *Beta* standardised slope, *p*^b^ *p* value of Wald test result

^c^Manageable workload

### Socio-demographic and labour features and the AWLS

As it can be seen (Table 5), 247 participants were men (54.6%). The participants showed a mean age of 36.6 years (SD = 10.03; range 18–60), in a stable relationship (75.6%), and having children (79.1%). 37.6% of them were working in a restaurant and 62.4% in a hotel, being 31.4% managers and 68.6% workers, and 72.2% had a permanent contract.

**Table 5:** Characteristics of participants (*n* = 452)

Sex*, male*	247 (54.6%)
Age^a^ *(range = 8–60)*	36.6 (10.03)
Relationships*, no*	110 (24.4%)
Children*, none*	95 (20.9%)
Business	
Restaurant	170 (37.6%)
Hotel	282 (62.4%)
Level	
Employee	403 (89.2%)
Manager	49 (10.8%)
Contract	
Permanent	326 (72.2%)
Temporary	126 (27.8%)
Stress symptoms^a^ *(range = 0–30)*	5.32 (3.88)
Giving-up ideas*, no*	387 (85.5%)
Absenteeism*, no*	337 (74.6%)

The rest of values are frequencies (percentages)

^a^Mean (SD)

Likewise, Table 6 shows the raw and adjusted ORs for the AWLS components according to socio-demographic and work characteristics. The high scores in each of the AWLS indicate a better relationship with work. Regarding manageable workload, those participants who had a manager level were less likely to have a higher score than those who worked as employees (adjusted OR = 0.28; 95% CI = 0.10–0.78); those participants who had a temporary contract were less likely to have a higher score than those who had a permanent contract (adjusted OR = 0.45; 95% CI = 0.21–0.96); and those participants with higher levels of psychosomatic symptoms were related to lower scores (adjusted OR = 0.83; 95% CI = 0.73–0.94). Concerning control, those participants who had a manager level were more likely to have a higher score than those who worked as employees (adjusted OR = 2.08; 95% CI = 1.25–3.48); those employees with higher symptoms levels were related to lower scores (adjusted OR = 0.86; 95% CI = 0.79–0.94). As for reward, higher symptoms levels were related to lower scores (adjusted OR = 0.84; 95% CI = 0.75–0.93); those participants who had giving-up ideas showed a lower likelihood of having a higher score than those with no giving-up ideas (adjusted OR = 0.57; 95% CI = 0.40–0.83). Regarding community, those participants who were older (adjusted OR = 0.96; 95% CI = 0.93–0.98) and had more presence of symptoms (adjusted OR = 0.87; 95% CI = 0.80–0.94), showed a lower likelihood of having a high score. Concerning fairness, as in the case of reward, higher symptoms levels were related to lower scores (adjusted OR = 0.89; 95% CI = 0.82–0.97), and those participants who had giving-up ideas showed a lower likelihood of having a higher score than those with no giving-up ideas (adjusted OR = 0.56; 95% CI = 0.41–0.77). With regard to values, those participants who had a manager level were more likely to have a higher score than those who worked as employees (adjusted OR = 2.51; 95% CI = 1.16–5.45); higher symptoms levels were related to lower scores in values (adjusted OR = 0.92; 95% CI = 0.85–0.99); those participants who had giving-up ideas showed a lower likelihood of having a higher score in values than those with no giving-up ideas (adjusted OR = 0.55; 95% CI = 0.39–0.75).

**Table 6:** Asociations between the socio-demographic and occupational characteristics of participants and the AWS factors

Variables (*ref*.)	Sex (*male*)	Age *(continuous*)	Partner (*no*)	Children (*none*)	Business (*Restaurant*)	Level (*employee*)	Contract (*permanent*)	Stress symptoms (*continuous*)	Give-up (*no*)	Absenteeism (*no*)
Workload^a^										
raw	0.72 (0.45–1.16)	0.96 (0.94–0.98)	1.15 (0.66–1.99)	0.45 (0.23–0.89)	0.75 (0.47–1.20)	0.17 (0.08–0.35)	0.24 (0.15–0.40)	0.77 (0.70–0.85)	1.75 (0.97–3.17)	1.18 (0.63–2.23)
adj.	–	0.98 (0.94–1.02)	–	0.60 (0.26–1.42)	–	0.28 (0.10–0.78)	0.45 (0.21–0.96)	0.83 (0.73–0.94)	–	–
Control										
raw	1.13 (0.71–1.80)	1.00 (0.98–1.03)	1.67 (0.92–3.03)	1.35 (0.63–2.91)	0.47 (0.52–1.36)	1.80 (1.10–2.95)	0.81 (0.48–1.36)	0.91 (0.84–0.98)	1.12 (0.59–2.14)	1.78 (0.99–3.21)
adj	–	–	–	–	–	2.08 (1.25–3.48)	–	0.86 (0.79–0.94)	–	–
Reward										
raw	0.75 (0.43–1.30)	0.97 (0.94–0.99)	1.11 (0.57–2.15)	0.51 (0.24–1.10)	0.73 (0.42–1.25)	0.87 (0.48–1.59)	1.78 (0.99–3.18)	0.81 (0.73–0.90)	0.60 (0.43–0.83)	1.30 (0.63–2.69)
adj	–	0.96 (0.93–1.00)	–	–	–	–	1.59 (0.82–3.08)	0.84 (0.75–0.93)	0.57 (0.40–0.83)	–
Community										
raw	1.15 (0.75–1.78)	0.96 (0.94–0.99)	1.41 (0.82–2.44)	0.63 (0.32–1.25)	1.10 (0.70–1.74)	1.00 (0.63–1.62)	1.66 (1.04–2.67)	0.87 (0.81–0.94)	0.79 (0.41–1.52)	0.63 (0.34–1.17)
adj	–	0.96 (0.93–0.98)	–	–	–	–	1.26 (0.75–2.12)	0.87 (0.80–0.94)	–	–
Fairness										
raw	1.11 (0.71–1.74)	1.00 (0.98–1.03)	2.12 (1.15–3.93)	0.82 (0.42–1.62)	0.98 (0.62–1.55)	7.57 (3.65–15.72)	0.93 (0.56–1.55)	0.89 (0.82–0.95)	0.48 (0.36–0.64)	1.36 (0.77–2.41)
adj	–	–	1.41 (0.74–2.71)	–	–	1.60 (0.95–2.70)	–	0.89 (0.82–0.97)	0.56 (0.41–0.77)	–
Values										
raw	1.23 (0.79–1.93)	1.00 (0.98–1.03)	1.17 (0.67–2.03)	0.95 (0.48–1.90)	0.66 (0.42–1.05)	4.16 (2.05–8.44)	0.95 (0.56–1.61)	0.90 (0.84–0.97)	0.47 (0.35–0.63)	1.08 (0.59–1.97)
adj.	–	–	–	–	–	2.51 (1.16–5.45)	–	0.92 (0.85–0.99)	0.55 (0.39–0.75)	–

Values are odds ratio (95% confidence interval in brackets) from logistic regression models

*Raw* raw models, *Adj* adjusted models, *Ref* reference category

^a^Manageable workload

## Discussion

As Rich, Lepine and Crawford [14] recommend, future research on burnout or engagement at work should focus not that much on the specific characteristics of individuals, but on the relationship between existing demands in the work environment and the worker’s own resources [40]. In this sense, the Areas of Work Life model [41] focuses on the variables present in the work environment that may cause some mismatch degree between the individual and his or her job. Therefore, the main objective of this study was to analyze the predictive power of these Six Areas on the burnout dimensions: exhaustion, cynicism and lack of effectiveness.

As other studies have shown, the Spanish validated version of MBI-GS has solid psychometric properties (factorial structure, reliability and validity) [27, 42]. Likewise, it was possible to verify it in our study, in which the solution of three factors explained a high percentage of the variance and its three dimensions showed an adequate adjustment and a high internal consistency between them. The inter-factor latent correlations between the MBI-GS dimensions were very high between exhaustion and cynicism, and moderate both between exhaustion and inefficacy and between cynicism and inefficacy.

The AWLS questionnaire also showed adequate properties, since the six-factor solution explained an adequate percentage of the variance and presented a good fit to the data, with adequate internal consistency values.

The explanatory power of the six AWLS variables on the three burnout components was significant. Furthermore, the best explained burnout dimension was exhaustion, while cynicism was the least explained. Manageable workload turned out to be the AWLS component that most contributed to explaining exhaustion. Also, the manageable workload, along with the rewards variable, were the components that contributed to explaining the cynicism dimension. Finally, the efficacy dimension was explained by manageable workload, and also by the variables control, community and congruence of values. All these relationships coincide with the different investigations that have applied the Areas of Work Life model to try to explain the processes by which employees can present high levels of exhaustion, cynicisms, and inefficacy. Manageable load has been shown to be the variable that acts most directly on burnout; however, in several organizations a different pattern has also been found: despite bearing a high overload rate, data show low levels of burnout and cynicism and high efficiency, given the fact that other areas, such as control, rewards, fairness, community, and, especially, values (congruence between the own values and values of the organization) may be acting as protective variables, or as mediating variables [18]. These findings confirm what has been called the two-process model of burnout [43].

In our study, managers showed a lower probability of feeling their workload as something manageable, that is, they were more overburdened than the rest of the employees, but the former reported greater possibility of task control and also greater congruence between their own values and those of their company. It is logical to think that managers bear a higher level of overload, but in return they have more strategies and possibilities of task control, such as delegation or introducing changes, while employees have a null or limited maneuverability. It is important to note that the workload measure includes work extending to personal life. This is a big issue for managers who do not receive overtime pay when they work more hours.

Job instability was shown to be inversely related to a manageable workload. Age and, closely related to it, the number of years in the company, was positively related to the sense of community. On the other hand, the stress symptomatology perceived by employees and managers was related to a higher workload index, a lack of task control, less sense of justice and perceived rewards, and a higher index of incongruence between the own values and those of the organization. Likewise, this symptomatology was more likely to appear in those who considered the option of leaving work, as well as in older workers.

These data confirm that the AWLS model explains a high percentage of the variance referred to the three burnout dimensions, but obviously it does not account for the total of this burnout phenomenon, which should not be explained solely by the variables related to the organization. The data discourage the proposals from recent decades to move away from factors such as personality variables [44] or other traits [45] such as self-efficacy [46] to explain the burnout process.

## Conclusions

These results make a valuable contribution. On the one hand, it is essential to know the rates of professional attrition in an organization, and even more, to know which variables explain this data in a positive or negative way. On the other hand, when designing intervention and prevention programs, it is advisable, as the authors have pointed out, to rely on the strong points found in the organization to be able to further enhance them and, in this way, to act more directly on the most obvious mismatches. Prevention should focus precisely on enhancing those positive aspects and incorporating medium and long-term actions to strengthen the areas of working life and, therefore, change the direction of the dimensions of exhaustion, cynicism and effectiveness.

Intervention experiences over the last two decades have made significant progress, focusing only on one, or at most two areas, to work on for a period of one or 2 years. It has been found that the areas have a great relationship with each other. For example, it has been observed that, by encouraging fair decision-making in the company, the sense of community and communication increase and workers feel rewarded. Also, by establishing rewards dispensation with clear criteria, employees report better levels of fairness [47].

The results of this study confirm that the Spanish version of the AWLS questionnaire is a good model to evaluate areas of working life in the hostelry field, with a significant explanatory power over each of the burnout variables. Therefore, it could be used to verify that interventions are carried out as expected.

## Data Availability

The datasets used and analysed during the current study are available from the corresponding author on reasonable request.

## Abbreviations

AWLS: *Areas of Worklife Scale*
*MBI-GS*: *Maslach Burnout Inventory-General Survey*

## References

[1] Freudenberger HJ (1974). Staff burnout. J Soc Issues.

[2] Firth H, McIntee J, McKeown P, Britton P (1986). Burnout and professional depression: related concepts?. J Adv Nurs.

[3] Pines A, Aronson E (1988). Career burnout: causes and cures.

[4] Maslach C, Jackson SE (1981). The measurement of experienced burnout. J Organ Behav.

[5] Maslach C, Schaufeli WB, Leiter MP (2001). Job burnout. Annu Rev Psychol.

[6] Montgomery C, Rupp AA (2005). A meta-analysis for exploring the diverse causes and effects of stress in teachers. Can. J. Educ.

[7] Schaufeli WB, Leiter MP, Maslach C (2009). Burnout: 35 years of research and practice. Career Dev Int.

[8] Lonsdale C, Hodge K, Jackson SA. Athlete engagement: II. Development and initial validation of the athlete engagement questionnaire. Int J Sport Psychol. 2007;38(4):471–92. Retrieved from https://psycnet.apa.org/record/2008-02698-008.

[9] Schaufeli WB, Salanova M, González-Romá V, Bakker AB (2002). The measurement of engagement and burnout: a two sample confirmatory factor analytic approach. J Happiness Stud.

[10] Maslach C, Leiter MP (1997). The truth about burnout.

[11] Leiter MP, Schaufeli WB (1996). Consistency of the burnout construct across occupations. Anxiety Stress Coping.

[12] Schaufeli WB, Dierendonck DV, Gorp KV (1996). Burnout and reciprocity: towards a dual-level social exchange model. Work & Stress.

[13] Schaufeli WB, Bakker AB (2003). Utrecht work engagement scale: preliminary manual.

[14] Rich BL, Lepine JA, Crawford ER (2010). Job engagement: antecedents and effects on job performance. Acad Manag J.

[15] Leiter MP, Maslach C. Preventing burnout and building engagement: team member's workbook. San Francisco: Jossey-Bass; 2000. ISBN: 0-7879-5539-6

[16] Landsbergis PA (1988). Occupational stress among health care workers: a test of the job demands-control model. J Organ Behav.

[17] Nishimura Kunihiro, Nakamura Fumiaki, Takegami Misa, Fukuhara Schunichi, Nakagawara Jyoji, Ogasawara Kuniaki, Ono Junichi, Shiokawa Yoshiaki, Miyachi Shigeru, Nagata Izumi, Toyoda Kazunori, Matsuda Shinya, Kataoka Hiroharu, Miyamoto Yoshihiro, Kitaoka Kazuyo, Kada Akiko, Iihara Koji (2014). Cross-Sectional Survey of Workload and Burnout Among Japanese Physicians Working in Stroke Care. Circulation: Cardiovascular Quality and Outcomes.

[18] Leiter MP, Gascón S, Martínez-Jarreta B (2010). Making sense of work life: a structural model of burnout. J Appl Soc Psychol.

[19] Bowers L, Nijman H, Simpson A, Jones J (2011). The relationship between leadership, teamworking, structure, burnout and attitude to patients on acute psychiatric wards. Soc Psychiatry Psychiatr Epidemiol.

[20] Riolli L, Savicki V (2006). Impact of fairness, leadership, and coping on strain, burnout, and turnover in organizational change. Int J Stress Manag.

[21] Leiter MP, Harvie P (1997). Correspondence of supervisor and subordinate perspectives during major organizational change. J Occup Health Psychol.

[22] Walsh WB, Craik KH, Price RH (1992). Person-environmental psychology: models and perspectives.

[23] Runhaar P, Konermann J, Sanders K (2013). Teachers’ organizational citizenship behaviour: considering the roles of their work engagement, autonomy and leader–member exchange. Teach Teach Educ.

[24] Sciberras A, Pilkington L (2018). The lived experience of psychologists working in mental health services: an exhausting and exasperating journey. Prof Psychol Res Pract.

[25] Cuadrado Roura JR, López Morales JM (2015). El turismo, motor del crecimiento y de la recuperación de la economía española.

[26] Kline RB (2011). Principles and practice of structural equation modeling.

[27] Maslach C, Jackson SE, Leiter MP (2017). Maslach burnout inventory manual.

[28] Faúndez VEO, Gil-Monte PR (2009). Análisis de las principales fortalezas y debilidades del “Maslach burnout inventory”(Mbi). Ciencia Trabajo.

[29] Gascón S, Leiter MP, Stright N, Santed MA, Montero-Marín J, Andrés E (2013). A factor confirmation and convergent validity of the “areas of worklife scale”(AWS) to Spanish translation. Health Qual Life Outcomes.

[30] Muthen B, Kaplan D (1992). A comparison of some methodologies for the factor analysis of non-normal Likert variables: a note on the size of the model. Br J Math Stat Psychol.

[31] Jöreskog K, Enslein K, Ralston A, Wilf HS (1977). Factor analysis by least-squares and maximum-likelihood methods. Statistical Methods for Digital Computers.

[32] Briggs NE, Maccallum RC (2003). Recovery of weak common factors by maximum likelihood and ordinary least squares estimation. Multivariate Behav Res.

[33] Byrne BM (2001). Structural equation modeling with Amos: basic concepts, applications and programming.

[34] Hu LT, Bentler PM (1999). Cutoff criteria for fit indexes in covariance structure analysis: conventional criteria versus new alternatives. Struct Equ Modeling.

[35] Lévy JP, Martín MT, Román MV, Lévy JP, Varela J (2006). Optimización según estructuras de covarianzas. Modelización con Estructuras de Covarianzas en Ciencias Sociales.

[36] Bollen K (1986). Sample size and bentler and Bonett’s nonnormed fit index. Psychometrika.

[37] McDonald RP (1999). Test theory: a unified treatment.

[38] Field A (2017). Discovering statistics using SPSS.

[39] Montero-Marin J, García-Campayo J, Fajó-Pascual M, Carrasco JM, Gascón S, Gili M, Mayoral-Cleries F (2011). Sociodemographic and occupational risk factors associated with the development of different burnout types: the cross-sectional University of Zaragoza study. BMC Psychiatry.

[40] Taris TW, Ybema JF, van Beek I (2017). Burnout and engagement: identical twins or just close relatives?. Burn Res.

[41] Leiter MP, Maslach C (1999). Six areas of worklife: a model of the organizational context of burnout. J Health Hum Serv Adm.

[42] Aguayo R, Vargas C, de la Fuente EI, Lozano LM. A meta-analytic reliability generalization study of the Maslach burnout inventory. Int J Clin Health Psychol. 2011;11(2):343-61. ISSN 1697-2600.

[43] Leiter MP, Gascón S, Martínez-Jarreta B (2008). A two process model of burnout: their relevance to Spanish and Canadian nurses. Psychol Spain.

[44] Mojsa-Kaja J, Golonka K, Marek T (2015). Job burnout and engagement among teachers - Worklife areas and personality traits as predictors of relationships with work. Int J Occup Med Environ Health.

[45] Zhang Y, Gan Y, Cham H (2007). Perfectionism, academic burnout and engagement among Chinese college students: a structural equation modeling analysis. Personal Individ Differ.

[46] Halbesleben JRB (2010). A meta-analysis of work engagement: relationships with burnout, demands, resources, and consequences.

[47] Awa WL, Plaumann M, Walter U (2010). Burnout prevention: a review of intervention programs. Patient Educ Couns.

